# Electronic Cigarette Use in Students and Its Relation with Tobacco-Smoking: A Cross-Sectional Analysis of the i-Share Study

**DOI:** 10.3390/ijerph14111345

**Published:** 2017-11-05

**Authors:** Shérazade Kinouani, Edwige Pereira, Christophe Tzourio

**Affiliations:** 1 Team HEALTHY, Bordeaux Population Health Research Center, University of Bordeaux, Inserm, UMR 1219, CHU Bordeaux, F-33000 Bordeaux, France; edwige.pereira@u-bordeaux.fr (E.P.); christophe.tzourio@u-bordeaux.fr (C.T.); 2 Department of General Practice, University of Bordeaux, 146 rue Léo Saignat F-33000 Bordeaux, France

**Keywords:** electronic cigarettes, smoking, young adult

## Abstract

While young adults often try e-cigarettes, little is known about its use and the reasons for experimentation, particularly in relation with tobacco-smoking. In 2016, data were collected from 2720 French-speaking students participating in a web-based study on students’ health: the internet-based Students Health Research Enterprise (i-Share) project. Univariate analyses and multivariable logistic regressions were performed to study the relationship between e-cigarette use and smoking status. Two out of five students declared having tried e-cigarettes and 3.6% were current users. Former smokers were more likely than current smokers to use e-cigarettes currently. Among those who had never smoked, 13.5% had tried e-cigarettes. Very few (0.3%) were current users, alternating e-liquids with and without nicotine. The three main reasons for trying e-cigarettes were curiosity, offer to try by someone, and attractiveness of e-liquid flavors. Among current smokers, previous attempts to quit smoking and a strong desire to stop tobacco were reported more in e-cigarette current users than in former users. In this large sample of French students, findings were consistent with the possibility that e-cigarettes might be used as smoking cessation or reduction aids by some young adults whereas other young never-smokers could be exposed to nicotine.

## 1. Introduction

Electronic cigarettes (or e-cigarettes) are electronic devices that can deliver nicotine in aerosol form without burning tobacco. Their use is increasing worldwide [1,2,3,4] and is more common among current smokers and former smokers than never-smokers [5,6,7,8]. The prevalence of e-cigarette use differs between countries, according to national policies. In France, 26% of 15–75 years old had tried e-cigarettes in 2014; 3% were daily users [9]. Their promotion and sale are partially regulated in France. The liquid that is vaporized is considered neither to be a tobacco product nor a medicinal drug but as a product for regular consumption that can be sold only in specialist shops. However, its sale is forbidden to people under the age of 18. Advertising for electronic devices is prohibited as is their use in certain places like schools and closed forms of public transport.

In Europe, smoking prevalence in 2014 was higher among adults aged 25–39 years (33%) or 40–54 years (34%) than in subjects aged 15 to 24 years (25%) or 55 and over (17%) [10]. Smoking prevalence in European young adults was higher than in the U.S. population for the same age [11]. Nicotine replacement therapy and electronic cigarettes were the most common tools used to stop tobacco without assistance by European people [10]. Indeed, the most frequently reported reason for e-cigarette use in previous studies was smoking reduction or cessation even though its safety and efficacy as a support to smoking cessation are still under study [2,12]. Furthermore, it is mainly adolescents and young adults who use them [7,13,14,15]. While several studies have explored the beliefs and perceptions of young populations about e-cigarettes [16] or their awareness related to their harmfulness [17,18], few have focused specially on the reasons for using them. In a survey of adults in the USA in 2013, the reasons for experimenting with e-cigarettes differed with age [19]. Among people 18–34 years old, the most common reason was curiosity. The frequency of this reason decreased with age, the most frequent reason for trying in those aged 55 being the desire to reduce or stop smoking.

Since tobacco and e-cigarette use levels were both high in young adults, we wondered whether they tried e-cigarettes for reasons related or not with smoking (cessation or decrease). We also wanted to establish the characteristics of young smokers who use e-cigarettes, in terms of tobacco addiction and motivation to quit smoking. We, therefore, sought to find out which young adults had tried e-cigarettes and why. The main objective of this study was to describe the relationship between electronic cigarette use and tobacco-smoking in a large sample of French students. Another objective was to describe the reasons for experimenting with e-cigarettes in these young adults. As with several previous studies which were conducted in student populations [20,21,22,23,24], we chose to recruit volunteers to participate in an online survey. This allowed us to quickly collect data among the target population.

## 2. Materials and Methods

### 2.1. Participants and Data Collection

We carried out the current study in a sample of students who already participated to a research project: the i-Share project (internet-based Students Health Research Enterprise). This project (www.i-share.fr) is an ongoing prospective e-cohort study of French-speaking students which started to recruit in February 2013. It is supported by three main French universities: the Universities of Bordeaux, Versailles, and Nice. In these partner universities, students are contacted via their email, by communication in the classes, or on the campuses. However, students not belonging to the three partner universities can also participate in the study by filling the i-Share baseline questionnaire online. To participate in i-Share, volunteers had to be registered at a university or higher education institute and understand French. When they filled the baseline questionnaire, students provided their national identification number to confirm their student status. This is a personal number assigned to each student at their first registration in a French higher education institution. This number was not mandatory to participate in the i-Share study in order to allow to students outside France to participate if they wished. The i-Share baseline questionnaire collected information about sociodemographic characteristics, academic characteristics, health status, personal and family medical history, and lifestyle habits.

**Figure 1:**
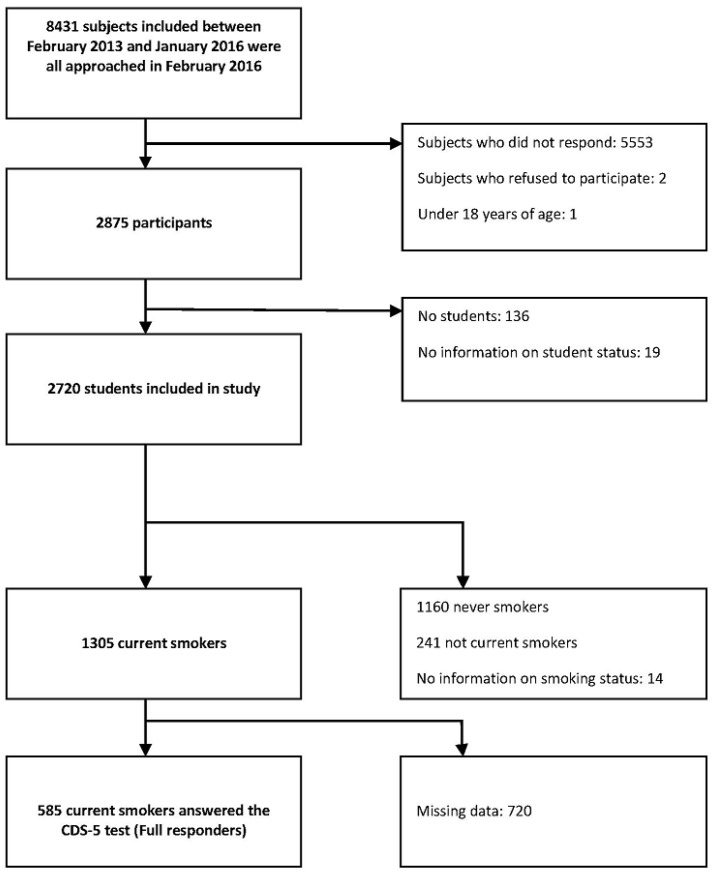
Flow chart. CDS-5: 5-item Cigarette Dependence Scale.

All 8431 students who completed the i-Share baseline questionnaire in February 2016 were eligible for this survey on e-cigarette use. Students were invited by e-mail to participate in a study on e-cigarette use nested in i-Share. We asked them to fill out a first questionnaire about e-cigarette use, and they were then invited to answer follow-up questionnaires every 6 months for 18 months. Our current analysis was conducted on data collected by the first e-cigarette questionnaire. They then received a recall by email once a week for three weeks if they did not fill it in. Overall, 2875 students completed the first questionnaire about e-cigarette use between February and April 2016 (response rate: 34.1%) and 2720 of them were retained for this analysis (Figure 1). We excluded from the analysis 136 subjects who were no longer students and 19 who did not specify their student status. Participants included and excluded are shown in Supplementary Materials (Tables S1 and S2).

All subjects gave their informed consent for inclusion before they participated in the i-Share project and its ancillary studies. The i-Share project was conducted in accordance with the Declaration of Helsinki. The protocol was approved by the CNIL (Commission Informatique et Libertés) which is the national authority that ensures that data collection in research does not violate freedoms, rights, and human privacy (number: DR-2013-019). The study protocol was evaluated by the Local Committee for the Protection of Persons of Bordeaux University which indicated that this research was outside the scope of the provisions governing biomedical research. Students participating in the study on e-cigarettes received some points in the i-Share study that can be exchanged for cinema tickets or fruit and vegetable hampers.

### 2.2. Measures

#### 2.2.1. Sociodemographic and Academic Measures

Data analyzed were: gender, age, academic year of study, university location, university major, educational level of parents, and income source. For university major, students were divided into five groups: sciences (computer sciences, mathematics, physics, biology, and sport sciences); healthcare (medicine, pharmacy, dentistry, and nursing); economics, management and law; literature, arts, humanities and social sciences (including languages and political sciences), and others.

#### 2.2.2. Electronic Cigarette Use

We used the definitions of the French Monitoring Centre for Drugs and Drug addiction (Observatoire français des drogues et des toxicomanies) [25] and the European Commission to explore e-cigarette use [9,10]. E-cigarette experimentation was defined as having tried an e-cigarette at least once. We distinguished three groups: (1) current users were those declaring the use of e-cigarettes daily or occasionally (less than once a day); (2) former users had tried e-cigarettes but were not using them at the time of the study; and (3) never users had never tried an e-cigarette. Current and former e-cigarette users specified the age (in years) when they first tried e-cigarettes in a non-mandatory question. They completed a 15-item questionnaire about their reasons for trying e-cigarettes (see Figure S1). We also asked current e-cigarette users if their e-liquid cartridges contained nicotine.

#### 2.2.3. Tobacco-Smoking

Regarding tobacco-smoking, we distinguished three groups: (1) current smokers were those smoking daily or occasionally (less than once a day); (2) former smokers had previously smoked tobacco but were no longer doing so; and (3) never smokers had never tried tobacco. If they wanted, current and former smokers specified their age (in years) when they tried smoking. When data were available, we calculated the number of years between trying e-cigarettes and trying tobacco. We categorized this continuous variable in three groups: (1) e-cigarette before tobacco; (2) e-cigarette and tobacco the same year; and (3) e-cigarette after tobacco. Current smokers filled in additional questions about their nicotine dependence, number of attempts to quit smoking, and desire to stop tobacco. Nicotine dependence was assessed with the 5-item Cigarette Dependence Scale (CDS-5), a scale assessing the likelihood of nicotine dependence in smokers out of a score of 25 [26,27]. The responses were analyzed as a continuous variable. We also asked current cigarette smokers “how many times have you tried to quit smoking?” and divided their responses into four classes: never/1 attempt/2 or 3 attempts/4 attempts or more. Finally, we asked them on a four-point scale whether they wanted to quit smoking: not at all/very little/somewhat/a lot. Among the 1305 current smokers included in the analysis, 585 agreed to answer these additional questions in detail. The comparison between these 585 smokers (called “full responders”) and those who did not answer is available in Supplementary Materials (Table S3).

### 2.3. Statistical Analysis

First, we described the sociodemographic and academic characteristics in the whole sample according to e-cigarette use. Then, we described e-cigarette use in the whole sample according to smoking status in univariate and multivariable analyses. Third, we analyzed the reasons for trying e-cigarettes among current and former e-cigarette users, stratified by smoking status. In the subgroup of 585 full responder smokers, we described nicotine dependence, attempts to stop smoking, and the desire to quit in relation to e-cigarette use.

We described continuous variables using median and inter-quartile range (IQR) and categorical variables using proportion. We then explored the factors associated with e-cigarette use by running univariate analyses. The Student test was used to analyze continuous variables and the Kruskal-Wallis test to compare the distribution of continuous variables in more than two independent samples. Univariate comparison of proportions was performed using the chi-square test. Fisher’s exact test was used when the theoretical count in cells was less than five. Multivariable analysis was performed by using a multinomial logistic regression model. All explanatory variables associated with e-cigarette use in univariate analyses were retained for the initial model if *p* < 0.20. The collinearity of the explanatory variables was studied because of the impact on the model’s stability. The full models after backward stepwise selection procedure contained the following explanatory variables: age in categories, gender, and university major. Due to the collinearity between variables, we kept age in categories and gender as confounders in the final models. All *p*-values were two-tailed and we considered *p* < 0.05 to be statistically significant. All analyses were performed with SAS^®^ version 9.4 (SAS Institute Inc., Cary, NC, USA).

## 3. Results

### 3.1. Sample Characteristics Regarding E-Cigarette Use

The sample characteristics are summarized in Table 1. The median age was 21 years, IQR: 19–22. More than 90% of the sample was between 18 and 24 years of age. More than 3/4 of the participants were women. They mainly were undergraduate students (76%). Fewer than half (46%) were on a healthcare course, and 54% had a paid job. There were 1086 students (40%) who had tried an e-cigarette. The median age of e-cigarette experimentation was 19 years, IQR: 18–21. Among these experimenters, 988 (91%) were former users and 98 (9%) were current users of e-cigarettes (Table 1). Current and former e-cigarette users were more frequently women than men. They also reported paid employment or being on a healthcare course more than those who never used it.

There were no statistically significant associations between e-cigarette use and age, academic year of study, university major, university location, or educational level of parents.

### 3.2. Association between Electronic Cigarette Use and Smoking Status

There was a statistically significant association between e-cigarette use and smoking status in univariate and multivariable analyses. More than four out of five non-smoker students had never tried an e-cigarette. Students who had never tried an e-cigarette were more common among never smokers (86%) than among current (42%) or former smokers (27%) (Table 2).

Nine out of ten students (89.4%) were smokers or former smokers before they tried e-cigarettes (Table 2). Among smokers and former smokers, 18 students (3%) had tried e-cigarettes before tobacco (Table 2). Two of them started e-cigarettes two years before tobacco; the other students used e-cigarettes one year before tobacco. It was impossible to specify the test sequence for 40 students who had tried both products in the same year.

Among never-smokers, 13.5% had tried an e-cigarette (Table 2). In more than nine out of ten cases, they had discontinued their use at the time of the survey. Only 0.3% of non-smokers were current e-cigarette users (Table 2), such use being exclusively less than once a day. These non-daily e-cigarette users alternated e-liquids with and without nicotine.

Both current and daily use of e-cigarettes were higher in former smokers than in current smokers, respectively: 12% versus 5% and 9% versus 2%, *p* < 10^−4^. In multivariable analyses, compared to former e-cigarette use, the probability of reporting current e-cigarette use was higher in former smokers than in current smokers or never smokers (Table 3).

### 3.3. Reasons for Trying E-Cigarettes

**Figure 2:**
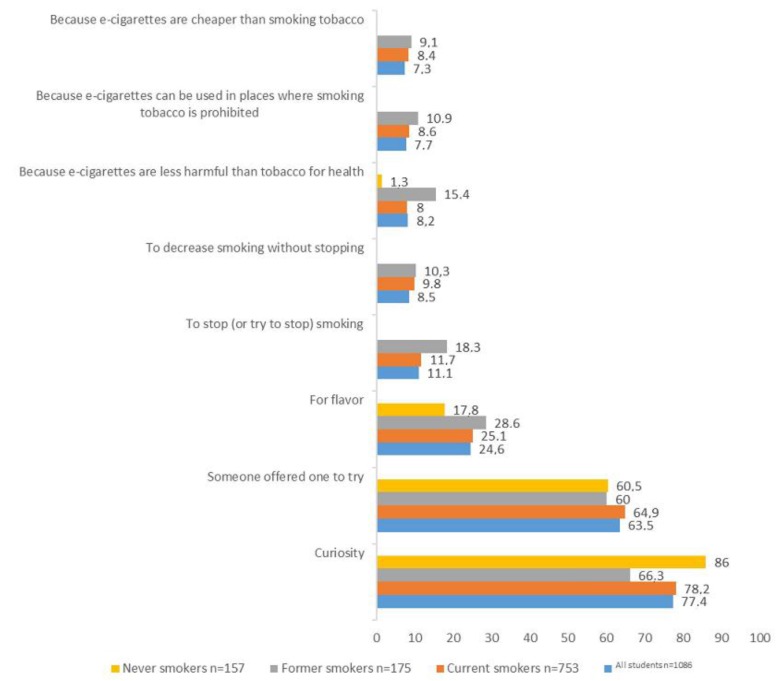
Reasons for trying e-cigarette (overall and according to smoking status) among students who had tried it. Missing data on smoking status, *n* = 1. Smoking status: current smokers were those smoking occasionally (less than once a day) or daily at time of survey. Former smokers were those who did not smoke at time of study.

The reasons for trying e-cigarettes were explored among students who had tried one at least once in their lives (*n* = 1086). They are summarized in Figure 2. The two main reasons were, in descending order of frequency, curiosity and someone offering one to try. There was no difference in the prevalence of these two main reasons between any of the categories of smoking status.

The attractiveness of flavor was placed third but at a much lower rate, while smoking cessation came only fourth among current and former smokers (Figure 2). Former smokers reported trying e-cigarettes to stop or decrease smoking more than current smokers: 29% versus 21%.

Men reported more than women that they had tried e-cigarettes in places where smoking tobacco is prohibited: 11% versus 6.5%, *p* = 0.0140. There were no other statistically significant associations between reasons for trying e-cigarettes and gender (Table 4).

### 3.4. Electronic Cigarette Use among Current Smokers

Among the 585 full responder smokers who participated in the study, 483 (83%) had tried e-cigarettes (Table 5). In this subgroup, 22% were currently trying to stop tobacco-smoking and this was more frequent in current e-cigarette users (43%) than in former users (19%). Current e-cigarette users had made more previous attempts to stop smoking than former or never users. They also reported more frequently a strong desire to stop smoking: 26% for current users and 11% for others (Table 5). The CDS-5 score did not differ for any category of smoking status (*p* = 0.08).

## 4. Discussion

In this study, two out of five students (40%) had tried an e-cigarette at least once, but only 9% remained current users. There was a statistically significant relationship between e-cigarette use and tobacco-smoking; experimentation as well as current or daily use of e-cigarette were more common in former smokers than in current smokers. Among never smokers, 13% had tried an e-cigarette. The three main reasons for trying e-cigarettes in decreasing order were curiosity, someone offering one, and the attraction of new flavors. In smokers, previous attempts to quit smoking and a strong desire to stop tobacco were reported more among current e-cigarette users than former users. There was no statistically significant association between e-cigarette use and nicotine dependence.

Most students who had tried an e-cigarette finally gave it up, whatever their smoking status. Almost nine in ten students were smokers or former smokers at least one year before trying an e-cigarette. In several previous studies in young people, e-cigarette experimentation as well as current use were more frequent in current tobacco smokers (called dual users) than in former smokers [17,28,29,30,31]. This was not the case in our study as former smokers were more likely current e-cigarette users than current smokers.

Thirteen percent of never-smokers had tried an e-cigarette but 97% of them had discontinued it at the time of the survey. A few of them continued to use it non-daily, alternating e-liquids with or without nicotine. Thus, a minority of never-smokers had an e-cigarette use which might have durably exposed them to nicotine. Although this was a minor phenomenon in our study, this exposure to nicotine by e-cigarette use could be a gateway into cigarette smoking. A recent meta-analysis investigated the initiation of tobacco smoking depending on the experimentation with e-cigarettes among adolescents and young adults [32]. The tobacco smoking initiation was more common among ever e-cigarette users than those who had never tried it.

The most common reason for trying an e-cigarette was curiosity. Findings suggested that never-smokers tried an e-cigarette mainly out of curiosity and then gave up it. To our knowledge, no study to date has specifically focused on motives of e-cigarette use in young adults who never smoked. However, our findings are similar to those found in never-smoker adolescents. For example, 2 to 5% of non-smoker adolescents in the United Kingdom had tried an e-cigarette in 2014 but fewer than 1% used it monthly [33]. Some studies in adolescents have shown that the intention to smoke cigarettes later was higher in non-smokers who had tried an e-cigarette than in non-smokers who had never tried one [34,35]. Longitudinal or qualitative research studies also are needed in young adults who do not smoke to understand why they use e-cigarettes and how this may accelerate the onset of tobacco-smoking.

We also found that previous attempts to quit smoking among current smokers were reported more by current users of e-cigarettes than by former or never e-cigarette users. They also more frequently reported being in the process of stopping smoking and having a stronger motivation for doing so. Thus, some of the smokers in our population might use e-cigarettes to change their tobacco consumption, which would be consistent with previous observations [14,36,37]. In absence of demonstrated efficiency of e-cigarettes in smoking cessation, caregivers must continue to propose to young adults some drugs indicated as a cessation aid: transdermal patches of nicotine, varenicline, and bupropion in France [38]. These treatments may be completed, where appropriate, with the use of e-cigarettes.

This cross-sectional analysis included a large number of participants. To our knowledge, this is the first study in French-speaking students who smoke to analyze the relationship between e-cigarette use and nicotine dependence or previous attempts to quit smoking. It is also the first study exploring the presence of nicotine in e-liquids among young French e-cigarette users.

This study had several limitations. First, the voluntary participation of students and the moderate response rate may have introduced a selection bias. Due to this voluntary participation, the constitution of our sample was quicker and easier. Nevertheless, as shown in the Supplementary Materials (Table S1), participants in the survey on e-cigarettes were more often female than male and were younger than those who did not participate. They went to Bordeaux campuses and trained in healthcare related studies more than non-participants. Thus, a selection bias was plausible. It was not also excluded that former smokers might have participated more than current or never-smokers in our study. All these might overestimate the prevalence of e-cigarette use in former smokers and underestimate it among never-smokers. Second, data available on the prevalence of tobacco-smoking among French-speaking young adults came from the Health Barometer 2010 survey [39]. This cross-sectional phone survey conducted among a representative sample of the French population including 6400 young people aged 15 to 30 years of age, 2276 of whom were students. This survey found that the prevalence of current smoking was 44% in 15–30-year-olds. However, that sample was not comparable to ours. Since we lacked recent data, it was impossible to know whether our proportions were close to reality. With a 48% prevalence of current smoking in our study, we cannot rule out an over-representation of smokers by self-selection bias. Representativeness and self-selection bias could have been partially managed by empirical or randomly selected sampling before the beginning of our study and by weighting during the data analysis. These management techniques of selection bias could not be applied because data about the characteristics of the target student population were not available. Third, the ratio of women to men in our sample was 4 to 1. This proportion of women was very high and remains incompletely explained. In France, women represented 57% of students enrolled in a university for the academic year 2015–2016 (Paris campuses: 58%; Bordeaux campuses: 56%, Nice campus: 55%, Versailles campuses: 52%) [40]. More than 75% of students included were French-speaking students enrolled in these four French universities. In 2016, women were overrepresented among the French undergraduate students in the most common majors in our study: Literature, arts, humanities and social (73.1%), healthcare (71.7%) [41]. Thus, our prevalence of female students could be explained by the female predominance in the university majors of the three main universities of i-Share (Bordeaux, Versailles, and Nice). It could also be due to a selection bias, with female students participating more often than male students in clinical research studies. In other recent studies in French student populations, there also was a female predominance [42,43] but in a lower rate. Fourth, analyses performed in the smokers' subgroup concerned only the 585 smokers who answered correctly about their smoking habits on the supplementary questionnaire. As shown in the Supplementary Materials (Table S3), these 585 smokers reported more smoking cessation being underway than the other smokers. Fifthly, some classification biases were plausible. All data were self-reported. It may be difficult to distinguish former and current smokers in a young population since students who smoke little or irregularly, as well as those who have stopped very recently, may find it difficult to define their smoking status. Similarly, the definition of former e-cigarette users in this cross-sectional analysis did not make it possible to distinguish those who stopped e-cigarette and tobacco consumption from those who stopped e-cigarette use but resumed tobacco-smoking. We collected age at start of tobacco smoking and of e-cigarette use. These questions were not mandatory and we had this information for only 550 students (59.3%) among 928 smokers/former smokers who had tried an e-cigarette at least once. It was impossible to specify the sequence for 40 students who started e-cigarette and tobacco the same year. Finally, former smokers reported more than current smokers that they had tried e-cigarettes to stop or decrease tobacco smoking in our study. However, a descriptive cross-sectional study did not allow for an evaluation of the direction of the association between e-cigarette use and smoking status. Former smokers could use the e-cigarette more than current smokers because they quit smoking after using e-cigarettes. They could also have quit smoking and then restarted using nicotine via the e-cigarettes to avoid smoking again. Therefore, caution is required when extrapolating our findings to other young populations.

## 5. Conclusions

The present findings suggest that the relationship between e-cigarette use and tobacco-smoking is complex among French-speaking students. Many students had tried an e-cigarette out of curiosity but did not continue using them. E-cigarette experimenters and current users were mainly former and current smokers. Smokers who currently used e-cigarettes (dual users) were often motivated to quit smoking and had tried to stop previously more often than had never or former e-cigarette users. These data were consistent with the possibility that e-cigarettes might be used as smoking cessation or reduction aids by young smokers. Nevertheless, a causality link was not found between the e-cigarette use and smoking cessation in our cross-sectional analyses. Additional longitudinal studies are needed to be able to determine the characteristics of young smokers who want to stop smoking and who would benefit from e-cigarette use as a stopgap measure. Two subgroups should have attention in further studies: young pregnant women who regularly use e-cigarettes and young continuous dual-users.

Very few never-smokers occasionally used e-cigarettes, sometimes by inhaling e-liquids with nicotine. There is also a need to understand the factors predicting the onset of tobacco-smoking in young adults who do not smoke but regularly use e-cigarettes. In the meantime, e-cigarettes should be not recommended for never-smokers.

## Figures and Tables

**Table 1:** Characteristics of study sample according to e-cigarette use.

Characteristic	E-Cigarette Use				*p* *
	All Students ^1^ *n* = 2712	Never Users ^2^ *n* = 1626	Former Users ^2^ *n* = 988	Current Users ^2^ *n* = 98	
	*n* (%)	*n* (%)	*n* (%)	*n* (%)	
Gender					<0.0001
Men	608 (22.4)	318 (19.6)	256 (25.9)	34 (34.7)	
Women	2104 (77.6)	1308 (80.4)	732 (74.1)	64 (65.3)	
Age in categories (years)					0.1979
18–20	1297 (47.8)	779 (47.9)	481 (48.7)	37 (37.8)	
21–24	1201 (44.3)	716 (44.0)	436 (44.1)	49 (50.0)	
25 and over	214 (7.9)	131 (8.1)	71 (7.2)	12 (12.2)	
Academic year of study					0.1251
First year	1105 (40.7)	655 (40.3)	409 (41.4)	41 (41.8)	
Second year	555 (20.5)	317 (19.5)	218 (22.1)	20 (20.4)	
Third year	409 (15.1)	238 (14.6)	152 (15.4)	19 (19.4)	
Fourth year or higher year of post-secondary education	643 (23.7)	416 (25.6)	209 (21.1)	18 (18.4)	
Universities and higher education institutes					0.0532
Bordeaux campuses	1657 (61.1)	970 (59.7)	630 (63.8)	57 (58.2)	
Nice campus	171 (6.3)	102 (6.3)	57 (5.8)	12 (12.2)	
Paris campuses	88 (3.2)	54 (3.3)	34 (3.4)	0 (0.0)	
Versailles campuses	278 (10.3)	173 (10.6)	92 (9.3)	13 (13.3)	
Other	518 (19.1)	327 (20.1)	175 (17.7)	16 (16.3)	
University major ^3^					0.0004
Sciences	294 (14.2)	180 (14.7)	103 (13.5)	11 (13.8)	
Healthcare	942 (45.6)	586 (47.7)	332 (43.7)	24 (30.0)	
Economics, management and law	188 (9.1)	97 (7.9)	78 (10.3)	13 (16.2)	
Literature, arts, humanities, and social sciences	412 (19.9)	214 (17.4)	177 (23.3)	21 (26.2)	
Other	232 (11.2)	151 (12.3)	70 (9.2)	11 (13.8)	
Educational level of parents ^4^					0.3784
Higher education study or university	1132 (43.7)	687 (44.3)	400 (42.5)	45 (46.9)	
High school	642 (24.8)	373 (24.1)	243 (25.8)	26 (27.1)	
Vocational study	748 (28.9)	444 (28.6)	282 (29.9)	22 (22.9)	
Primary education	66 (2.6)	46 (3.0)	17 (1.8)	3 (3.1)	
Income source					
Family ^5^					0.9942
Yes	2271 (90.8)	1376 (90.9)	815 (90.8)	80 (90.9)	
No	229 (9.2)	138 (9.1)	83 (9.2)	8 (9.1)	
Scholarship ^6^					0.1590
Yes	1056 (55.6)	649 (56.5)	379 (55.0)	28 (44.4)	
No	844 (44.4)	499 (43.5)	310 (45.0)	35 (55.6)	
Paid employment ^7^					<0.0001
Yes	1049 (54.4)	588 (50.4)	415 (60.1)	46 (63.9)	
No	879 (45.6)	578 (49.6)	275 (39.9)	26 (36.1)	

* Chi^2^-test. Missing data: ^1^ *n* = 8 (data missing on e-cigarette use); ^2^ Electronic cigarette use: never e-cigarette users were those who had never tried it. Current users comprised those regularly using e-cigarettes at time of survey, daily, or occasionally (less of once a day). Former users were those who were not current smokers among experimenters, as opposed to current users. ^3^ *n* = 652; ^4^ *n* = 132; ^5^ *n* = 220; ^6^ *n* = 820; ^7^ *n* = 792.

**Table 2:** E-cigarette use according to smoking status.

	Tobacco-Smoking ^1^				*p* *
	All Students ^2^ *n* = 2706	Never Smokers *n* = 1160	Former Smokers *n* = 241	Current Smokers *n* = 1305	
	*n* (%)	*n* (%)	*n* (%)	*n* (%)	
E-cigarette use ^3^					<0.0001
Never use	1621 (59.9)	1003 (86.5)	66 (27.4)	552 (42.3)	
Former use	987 (36.5)	153 (13.2)	146 (60.6)	688 (52.7)	
Current use	98 (3.6)	4 (0.3)	29 (12.0)	65 (5.0)	
Test sequence of tobacco and e-cigarette ^4,5^	*n* = 550		*n* = 80	*n* = 470	0.0009
E-cigarette before tobacco	18 (3.3)	-	3 (3.7)	15 (3.2)	
E-cigarette and tobacco the same year	40 (7.3)	-	6 (7.5)	34 (7.2)	
E-cigarette after tobacco	492 (89.4)	-	71 (88.8)	421 (89.6)	
Nicotine in cartridges ^6^	*n* = 98	*n* = 4	*n* = 29	*n* = 65	0.0152 ^γ^
Only e-liquids with nicotine	7 (7.1)	0 (0.0)	1 (3.5)	6 (9.2)	
Only nicotine-free e-liquids	61 (62.3)	0 (0.0)	17 (58.6)	44 (67.7)	
Both	19 (19.4)	4 (100.0)	6 (20.7)	9 (13.4)	
Unaware of nicotine in e-liquids	11 (11.2)	0 (0.0)	5 (17.2)	6 (9.2)	

* Chi^2^- test and ^γ^ Fisher’s exact test. Missing data: ^1^ Smoking status: current smokers were those smoking occasionally (less than once a day) or daily at time of survey. Former smokers were those who did not smoke at time of study; ^2^ *n* = 14 (data missing on smoking status); ^3^ Electronic cigarette use: never e-cigarette users were those who had never tried it. Current users comprised those regularly using e-cigarettes at time of survey, daily, or occasionally (less of once a day). Former users were those who were not current smokers among experimenters, as opposed to current users; ^4^ *n* = 378 (data missing on age, in years, at which students tried tobacco and/or e-cigarettes); ^5^ Test sequence of tobacco and e-cigarette: explored in smokers and former smokers. Chi-2 test about difference between smokers and former smokers; ^6^ Nicotine in cartridges: this question was asked only to current users of e-cigarette (*n* = 98).

**Table 3:** Associations between the e-cigarette use and smoking status.

Comparisons Depending on Smoking Status	Current E-Cigarette Use ^1^		
	Model 1 ^2^	Model 2 ^3^	Model 3 ^4^
	OR (95% CI)	AOR (95% CI)	AOR (95% CI)
Current smokers versus former smokers	0.48 (0.30–0.76)	0.50 (0.31–0.81)	0.51 (0.30–0.87)
Current smokers versus never smokers	3.61 (1.30–10.07)	3.62 (1.3–10.10)	4.12 (1.26–13.44)
Former smokers versus never smokers	7.60 (2.61–22.14)	7.26 (2.48–21.20)	8.11 (2.37–27.80)

AOR: adjusted odds ratios. OR: odds ratios. 95% CI: confidence interval to 95%. ^1^ Reference class: former e-cigarette use. ^2^ Logistic multinomial regression, without adjustment. ^3^ Logistic multinomial regression, adjusted for age in categories and gender (final models). ^4^ Logistic multinomial regression, adjusted for age in categories, gender, and university major (full models).

**Table 4:** Reasons for trying e-cigarette (overall and according to gender) among students who had tried it.

Reasons	All Students *n* = 1086	Gender		
		Men *n* = 290	Women *n* = 796	*p* *
	*n* (%)	*n* (%)	*n* (%)	
Curiosity	841 (77.4)	221 (76.2)	620 (77.9)	0.5573
Someone offered one to try	690 (63.5)	178 (61.4)	512 (64.3)	0.3728
For flavor	267 (24.6)	60 (20.7)	207 (26.0)	0.0719
To stop (or try to stop) smoking	120 (11.1)	39 (13.4)	81 (10.2)	0.1281
To decrease smoking without stopping	92 (8.5)	28 (9.7)	64 (8.0)	0.3978
Because e-cigarettes are less harmful than tobacco for health	89 (8.2)	30 (10.3)	59 (7.4)	0.1190
Because e-cigarettes can be used in places where smoking tobacco is prohibited	84 (7.7)	32 (11.0)	52 (6.5)	0.0140
Because e-cigarettes are cheaper than smoking tobacco	79 (7.3)	26 (9.0)	53(6.7)	0.1953

* Chi2-test.

**Table 5:** Characteristics of “full responder” smokers according to e-cigarette use.

Characteristic	E-Cigarette Use				*p* *
	Current Tobacco Smokers *n* = 585	Never Users ^1^ *n* = 102	Former Users ^1^ *n* = 430	Current Users ^1^ *n* = 53	
	*n* (%)	*n* (%)	*n* (%)	*n* (%)	
Gender					0.2504
Men	136 (23.3)	21 (20.6)	98 (22.8)	17 (32.1)	
Women	449 (76.7)	81 (79.4)	332 (77.2)	36 (67.9)	
Age in categories (years)					0.2588
18–20	271 (46.3)	48 (47.1)	205 (47.7)	18 (34.0)	
21–24	270 (46.2)	44 (43.1)	197 (45.8)	29 (54.7)	
25 and over	44 (7.52)	10 (9.8)	28 (6.5)	6 (11.3)	
CDS-5 ^2^ score, median (IQR) ^3^	9.0 (9.0, 11.0)	9.0 (9.0, 10.0)	9.0 (8.0, 11.0)	10.0 (8.0, 11.0)	0.0848 ^†^
Currently trying to stop tobacco-smoking					0.0003
No	457 (78.1)	80 (78.4)	347 (80.7)	30 (56.6)	
Yes	128 (21.9)	22 (21.6)	83 (19.3)	23 (43.4)	
Number of previous attempts to quit tobacco ^4^					0.0429
Never	254 (44.4)	56 (56.6)	181 (43.1)	17 (32.1)	
1 attempt	113 (19.8)	17 (17.2)	84 (20.0)	12 (22.6)	
2 or 3 attempts	157 (27.4)	23 (23.2)	118 (28.1)	16 (30.2)	
4 attempts or more	48 (8.4)	3 (3.0)	37 (8.8)	8 (15.1)	
Desire to stop smoking ^4^					0.0028
Not at all	131 (22.9)	31 (31.3)	92 (21.9)	8 (15.1)	
Very little	178 (31.1)	20 (20.2)	143 (34.1)	15 (28.3)	
Medium	193 (33.7)	37 (37.4)	140 (33.3)	16 (30.2)	
Strong	70 (12.3)	11 (11.1)	45 (10.7)	14 (26.4)	

Full responders: current smokers who filled in supplementary items about nicotine dependence, number of attempts to quit tobacco and desire to stop smoking. * Chi2-test, except: ^†^: Kruskal-Wallis test. Abbreviations: ^1^ Electronic cigarette use: never e-cigarette users were those who had never tried one. ^2^ CDS-5: 5-item Cigarette Dependence Scale; ^3^ IQR: interquartile range. ^4^ Missing data: *n* = 13. Current users comprised those regularly using e-cigarettes at time of survey, daily, or occasionally (less than once a day). Former users were those who were not current smokers among experimenters, as opposed to current users.

## Supplementary Materials

The following are available online at www.mdpi.com/1660-4601/14/11/1345/s1. Figure S1: Questions about e-cigarette use and reasons for experimenting with it, Table S1: Comparison between students who participated in the e-cigarette survey and those who did not, Table S2: Responders included in analysis about e-cigarette use and those excluded, Table S3: Smokers who responded on supplementary tests and those who did not.
