# Supplementary material for: Electronic Cigarette Use in Students and Its Relation with Tobacco-Smoking: A Cross-Sectional Analysis of the i-Share Study
Source: Int J Environ Res Public Health. 2017 Nov 5;14(11):1345. doi: 10.3390/ijerph14111345 (PMC5707984; doi:10.3390/ijerph14111345)
Supplement: Supplementary file 1 [file ijerph-14-01345-s001.pdf]

Table S1. Comparison between students who participated in the e-cigarette survey and those who did not.

	Guest students n=8431	No Participants n=5556	Participants n=2875	*p**
	n (%)	n (%)	n (%)	
Gender				0,0002
Men	2093 (24.8)	1450 (26.1)	643 (22.4)	
Women	6338 (75.2)	4106 (73.9)	2232 (77.6)	
Age in categories (years)				<0.0001
18 – 20	3549 (42.1)	2227 (40.1)	1322 (46.0)	
21 – 24	3885 (46.1)	2612 (47.0)	1273 (44.3)	
25 and over	997 (11.8)	717 (12.9)	280 (9.7)	
Academic year of study				0.1184
First year	3462 (41.0)	2331 (42.0)	1131 (39.3)	
Second year	1626 (19.3)	1053 (18.9)	573 (19.9)	
Third year	1254 (14.9)	824 (14.8)	430 (15.0)	
Fourth year or higher year of post-secondary education	2089 (24.8)	1348 (24.3)	741 (25.8)	
Universities and higher education institutes location				0.0001
Bordeaux campuses	5017 (59.5)	3242 (58.3)	1775 (61.7)	
Nice campus	589 (7.0)	413 (7.4)	176 (6.1)	
Paris campuses	212 (2.5)	120 (2.2)	92 (3.2)	
Versailles campuses	935 (11.1)	645 (11.6)	290 (10.1)	
Other	1678 (19.9)	1136 (20.5)	542 (18.9)	
University major [1]				<0.0001
Sciences	1054 (16.5)	737 (17.5)	317 (14.4)	
Healthcare	2209 (34.5)	1222 (29.1)	987 (44.9)	
Economics, management and law	768 (12.0)	575 (13.7)	193 (8.8)	
Literature, arts, humanities, and social sciences	1647 (25.7)	1193 (28.4)	454 (20.6)	
Other	722 (11.3)	474 (11.3)	248 (11.3)	
Educational level of parents [2]				0.0002
Higher education study or university	3267 (40.8)	2077 (39.5)	1190 (43.3)	
High school	2158 (27.0)	1477 (28.1)	681 (24.8)	
Vocational study	2308 (28.9)	1507 (28.7)	801 (29.2)	
Primary education	266 (3.3)	193 (3.7)	73 (2.7)	
Income source				
Family [3]				<0.0001
Yes	6843 (87.5)	4441 (86.0)	2402 (90.5)	
No	977 (12.5)	725 (14.0)	252 (9.5)	
Scholarship [4]				<0.0001
Yes	3174 (49.9)	2058 (47.5)	1116 (55.2)	
No	3183 (50.1)	2278 (52.5)	905 (44.8)	
Paid employment [5]				0.0002
Yes	3383 (51.8)	2243 (50.3)	1140 (55.2)	
No	3144 (48.2)	2219 (49.7)	925 (44.8)	

*Chi2 test. Missing data: [1] n=2031; [2] n=432; [3] n=611; [4] n=2074; [5] n=1904.

Table S2. Responders included in analysis about e-cigarette use and those excluded.

	All responders n=2875	Subjects excluded [6] n=155	Subjects included [6] n=2720	*p**
	n (%)	n (%)	n (%)	
Gender				0.4675
Men	643 (22.4)	31 (20.0)	612 (22.5)	
Women	2232 (77.6)	124 (80.0)	2108 (77.5)	
Age in categories (years)				<0.0001
[18 – 20]	1322 (46.0)	21 (13.5)	1301 (47.8)	
[21 – 24]	1273 (44.3)	68 (43.9)	1205 (44.3)	
[25] and over	280 (9.7)	66 (42.6)	214 (7.9)	
Academic year of study				<0.0001
First year	1131 (39.3)	24 (15.5)	1107 (40.7)	
Second year	573 (19.9)	18 (11.6)	555 (20.4)	
Third year	430 (15.0)	17 (11.0)	413 (15.2)	
Fourth year or higher year of post-secondary education	741 (25.8)	96 (61.9)	645 (23.7)	
Universities and higher education institutes				0.0347
location	1774 (61.7)	113 (72.9)	1661 (61.1)	
Bordeaux campuses	175 (6.1)	4 (2.6)	171 (6.3)	
Nice campus	90 (3.1)	2 (1.3)	88 (3.2)	
Paris campuses	290 (10.1)	12 (7.7)	278 (10.2)	
Versailles campuses	546 (19.0)	24 (15.5)	522 (19.2)	
Other				
University major [1]				0.0096
Sciences	317 (14.4)	23 (18.1)	294 (14.2)	
Healthcare	987 (44.9)	45 (35.4)	942 (45.4)	
Economics, management and law	193 (8.8)	5 (4.0)	188 (9.1)	
Literature, arts, humanities, and social	454 (20.6)	38 (29.9)	416 (20.1)	
sciences	248 (11.3)	16 (12.6)	232 (11.2)	
Other				
Educational level of parents [2]				0.1828
Higher education study or university	1190 (43.3)	57 (38.0)	1133 (43.7)	
High school	681 (24.8)	35 (23.3)	646 (24.9)	
Vocational study	801 (29.2)	51 (34.0)	750 (28.9)	
Primary education	73 (2.7)	7 (4.7)	66 (2.5)	
Income source				
Family [3]				0.0080
Yes	2402 (90.5)	123 (84.2)	2279 (90.9)	
No	252 (9.5)	23 (15.8)	229(9.1)	
Scholarship [4]				0.1213
Yes	1116 (55.2)	56 (48.3)	1060 (55.6)	
No	905 (44.8)	60 (51.7)	845 (44.4)	
Paid employment [5]				0.0117
Yes	1140 (55.2)	85 (65.9)	1055 (54.5)	
No	925 (44.8)	44 (34.1)	881 (45.5)	

*Chi2 test. Missing data: [1] n=676; [2] n=130; [3] n=221; [4] n=854; [5] n=810. [6] Students excluded: 136 subjects who were no longer students at time of survey and 19 subjects who did not specify their academic status.

Table S3. Smokers who responded on supplementary tests and those who did not.

	All smokers n=1305	Partial responders [6] n=720	Full responders [6] n=585	*p**
	n (%)	n (%)	n (%)	
Gender				0.8776
Men	306 (23.4)	170 (23.6)	136 (23.2)	
Women	999 (77.6)	550 (76.4)	449 (76.6)	
Age in categories (years)				0.2080
18 – 20	638 (48.9)	367 (51.0)	271 (46.3)	
21 – 24	579 (44.4)	309 (42.9)	270 (46.2)	
25 and over	88 (6.7)	44 (6.1)	44 (7.5)	
Academic year of study				0.1597
First year	545 (41.8)	295 (41.0)	250 (42.8)	
Second year	285 (21.8)	161 (22.4)	124 (21.2)	
Third year	188 (14.4)	93 (12.9)	95 (16.2)	
Fourth year or higher year of post-secondary education	287 (22.0)	171 (23.7)	116 (19.8)	
Universities and higher education institutes location				0.5979
Bordeaux campuses	816 (62.5)	440 (61.1)	376 (64.3)	
Nice campus	66 (5.1)	34 (4.7)	32 (5.5)	
Paris campuses	45 (3.4)	27 (3.8)	18 (3.1)	
Versailles campuses	132 (10.1)	79 (11.0)	53 (9.0)	
Other	246 (18.9)	140 (19.4)	106 (18.1)	
University major [1]				<0.0001
Sciences	130 (13.1)	71 (13.1)	59 (13.2)	
Healthcare	456 (46.1)	286 (52.8)	170 (38.0)	
Economics, management and law	104 (10.6)	48 (8.8)	56 (12.6)	
Literature, arts, humanities, and social sciences	209 (21.1)	86 (15.9)	123 (27.5)	
Other	90 (9.1)	51 (9.4)	39 (8.7)	
Educational level of parents [2]				0.2578
Higher education study or university	569 (45.3)	325 (46.9)	244 (43.3)	
High school	326 (26.0)	172 (24.8)	154 (27.4)	
Vocational study	338 (26.9)	187 (27.0)	151 (26.8)	
Primary education	23 (1.8)	9 (1.3)	14 (2.5)	
Income source				
Family [3]				0.0789
Yes	1098 (91.5)	616 (92.8)	482 (89.9)	
No	102 (8.5)	48 (7.2)	54 (10.1)	
Scholarship [4]				0.4230
Yes	502 (55.2)	261 (53.9)	241 (56.6)	
No	408 (44.8)	223 (46.1)	185 (43.4)	
Paid employment [5]				0.3163
Yes	534 (58.0)	281 (56.5)	253 (59.8)	
No	386 (42.0)	216 (43.5)	170 (40.2)	
Smoking cessation underway				<0.0001
No	1177 (90.2)	720 (100.0)	457 (78.1)	
Yes	128 (9.8)	0 (0.0)	128 (21.9)	

*Chi2 test. Missing data: [1] n= 316; [2] n=49; [3] n=105; [4] n=395; [5] n=385. [6] Full responders: current smokers who filled in supplementary items about their nicotine dependence, number of attempts to quit smoking and desire for stopping.

Figure S1. Questions about e-cigarette use and reasons for experimenting with it.

Question 1: Have you ever tried an electronic cigarette at least once in your lifetime? One possible answer
NO □ YES □

Question1.1: If « YES » to question 1: Why did you try it? Several possible answers
To stop (or try to stop) smoking □
To smoke less but without stopping □
Someone offered me one □
Because I received an e-cigarette as a gift □
Because my friends were smoking them □
Because you can smoke e-cigarettes in places where smoking is prohibited □
Because e-cigarettes are less harmful than tobacco for health □
Because smoking e-cigarettes are cheaper than smoking tobacco □
To never start smoking □
Out of curiosity □
Because of the flavors on offer □
To do the same as someone in my family □
To do the same as someone I know: friend or colleague □
I don't know □
Other reasons □

Question 2: If « YES » to question 1: Do you still use electronic cigarettes (occasionally or daily)? One possible answer
NO □ YES □

Question 2.1: If « YES » to question 2: How often do you use electronic cigarettes on average? One possible answer
Daily □
Less than once a day but at least once a week □
Less than once a week but at least once a month □
Less than once a month □
